# Improved thrombolytic effect with focused ultrasound and neuroprotective agent against acute carotid artery thrombosis in rat

**DOI:** 10.1038/s41598-017-01769-2

**Published:** 2017-05-09

**Authors:** Tsong-Hai Lee, Jih-Chao Yeh, Chih-Hung Tsai, Jen-Tsung Yang, Shyh-Liang Lou, Chen-June Seak, Chao-Yung Wang, Kuo-Chen Wei, Hao-Li Liu

**Affiliations:** 1 Stroke Center and Department of Neurology, Linkou Chang Gung Memorial Hospital and College of Medicine, Chang Gung University, Taoyuan, 333 Taiwan; 2 Departments of Electrical Engineering, Graduate Institute of Clinical Medical Sciences, Chang-Gung University, Taoyuan, 333 Taiwan; 3 Department of Neurosurgery, Chiayi Chang Gung Memorial Hospital and College of Medicine, Chang Gung University, Chiayi, Taiwan; 4 Department of Biomedical Engineering, Chung Yuan Christian University, Chung Li, Taiwan; 5 Department of Emergency Medicine, Linkou Chang Gung Memorial Hospital and College of Medicine, Chang Gung University, Taoyuan, 333 Taiwan; 6 Department of Cardiology, Linkou Chang Gung Memorial Hospital and College of Medicine, Chang Gung University, Taoyuan, 333 Taiwan; 7 Department of Neurosurgery, Linkou Chang Gung Memorial Hospital and College of Medicine, Chang Gung University, Taoyuan, 333 Taiwan; 8 Institute for Radiological Research, Chang Gung University/Chang Gung Memorial Hospital, Taoyuan, 333 Taiwan

## Abstract

Combination therapy with focused ultrasound (FUS) and a neuroprotective agent, BNG-1, was examined in an acute carotid thrombotic occlusion model using LED irradiation in rat to improve the thrombolytic effect of rt-PA. Seven treatment groups included (A) intravenous bolus injection of 0.45 mg/kg rt-PA, (B) intravenous bolus injection of 0.9 mg/kg, (C) sonothrombolysis with FUS alone, (D) oral administration of 2 g/kg BNG-1 for 7 days alone, (E) A + D, (F) A + C, and (G) A + C + D. Four comparison groups were made including (H) 0.45 mg/kg rt-PA 20% bolus +80% IV fusion + FUS, (I) 0.9 mg/kg rt-PA with 10% bolus + 90% intravenous fusion, (J) B + C, (K) B + D. At 7 days after carotid occlusion, small-animal carotid ultrasound and 7 T MR angiography showed the recanalization rate of ≤50% stenosis was 50% in group B and 83% in group I, but 0% in groups A and C and 17% in group D. Combination therapy improved recanalization rate to 50–63% in groups E and F, to 67–83% in groups J and K, and to 100% in groups G and H. Our study demonstrated combination therapy with different remedies can be a feasible strategy to improve the thrombolytic effect of rt-PA.

## Introduction

Stroke is known the second leading cause of death and the major cause of disability worldwide^1^. Intravenous recombinant tissue-type plasminogen activator (rt-PA) treatment has been used in acute ischemic stroke within 3–4.5 h after symptom onset since 1990s^2–4^. However, rt-PA thrombolysis is limited by slow reperfusion and carries significant risks of bleeding (10 folds higher than no rt-PA). Currently, there are strategies trying to improve the thrombolytic effect of rt-PA in acute major ischemic stroke such as intra-arterial mechanical thrombectomy, ultrasound enhancement (sonothrombolysis) and neuroprotective agents^5^.

Intra-arterial mechanical thrombectomy is announced in 2015 and is a new standard of care for patients with large-vessel strokes, which extends the therapeutic time window to 6 h or longer^6^. The recent 5 clinical trials have demonstrated the intra-arterial mechanical thrombectomy using a stent-retriever device plus intravenous rt-PA could be more effective when compared with intravenous rt-PA alone^7–11^. However, a recent meta-analysis showed although intra-arterial mechanical thrombectomy was associated with improved functional outcomes and higher rates of angiographic revascularization, there is still no significant difference in symptomatic intracranial hemorrhage or all-cause mortality at 90 days when compared to the standard intravenous rt-PA treatment^12^.

Sonothrombolysis used transcranial ultrasound to locally enhance the systemically acting rt-PA to accelerate fibrin clot dissolution, decrease rt-PA concentration required, increase localized fibrinolysis and decrease unwanted systemic fibrinolysis^13^. The effectiveness of sonothrombolysis using transcranial ultrasound has been investigated in a large phase III clinical trial (CLOTBUST-ER)^14^ and was found to be safe but had no significant benefit in acute stroke. Focused ultrasound (FUS) has been shown to be a much more effective method than transcranial ultrasound for blood clot lysis with or without thrombolytic drugs both *in vitro* and *in vivo* ^15–17^. Concentrated energy exposure of FUS can reduce the risk of potential off-target vascular damage when compared to planner unfocused ultrasound and can be applied transcranially as a noninvasive means to accelerate thrombolytic process in the context of acute ischemic stroke. Previous study showed that FUS-induced thrombolytic efficacy may depend on acoustic output power as well as other operating parameter combinations such as duty cycle and pulse width^18^. But generally, the overall thrombolysis rates can be benefited by FUS with the range from 10 to 42% in rabbit study^19^.

The BNG-1 is a formulation of traditional Chinese medicine^20^, consisting of eight herbs including Scutellaria Radix (6%), Angelica Radix (12%), Bupleuri Radix (14%), Coptis Rhizome (12%), Bambusa Concretio Silicea (16%), Glycyrrhiza Radix (12%), Ginseng Radix (14%) and Astragali Radix (14%). The phase II study of BNG-1 has shown safety with no increased bleeding risk when compared BNG-1 plus aspirin with aspirin alone^21^, and the phase III double-blind, randomized, placebo-controlled, multi-center study is now under investigation to compare the functional outcome and safety of treatment with BNG-1 in combination with aspirin with that of aspirin alone in ischemic stroke recovery (ClinicalTrials.gov identifier: NCT01675115; Taiwan DOH Protocol no.: BNG-TW-002, JIRB no.: 11-008-A). The BNG-1 has neuroprotective effect against ischemic injury which can be seen in animals receiving BNG-1 before as well as after middle cerebral artery (MCA) occlusion and has anti-thrombotic activity to inhibit arachidonic acid-induced platelet aggregation *in vitro* and prolong bleeding time in mice *in vivo* ^20^. Our previous study using 7T MR neuroimaging and neurotrophin analyses in rat MCA occlusion model^22^ found the neuroprotective effect of BNG-1 may act through recovery of cerebral hemodynamics, decrease of infarction size and edema, reduction of postischemic hyperperfusion injury and recovery of neurotrophn-3 and brain-derived neurotrophic factor.

Nowadays, intravenous rt-PA treatment is the standard treatment in most hospitals but is limited with small therapeutic time window. Intra-arterial mechanical thrombectomy can extend the therapeutic time window but is limited with high cost and relatively high risk. Also, it can be performed only in comprehensive stroke center with a well-trained team from different disciplines. The purpose of the present study intends to investigate a novel treatment strategy of combination therapy of rt-PA with FUS and BNG-1 for the recanalization of acute artery thrombotic occlusion, which can be easy to perform, costs less, reduces rt-PA dose and is able to extend the therapeutic time window to become an alternative therapy other than mechanical thrombectomy.

## Results

### Preliminary studies of lysis effect by focused ultrasound

**Figure 1:**
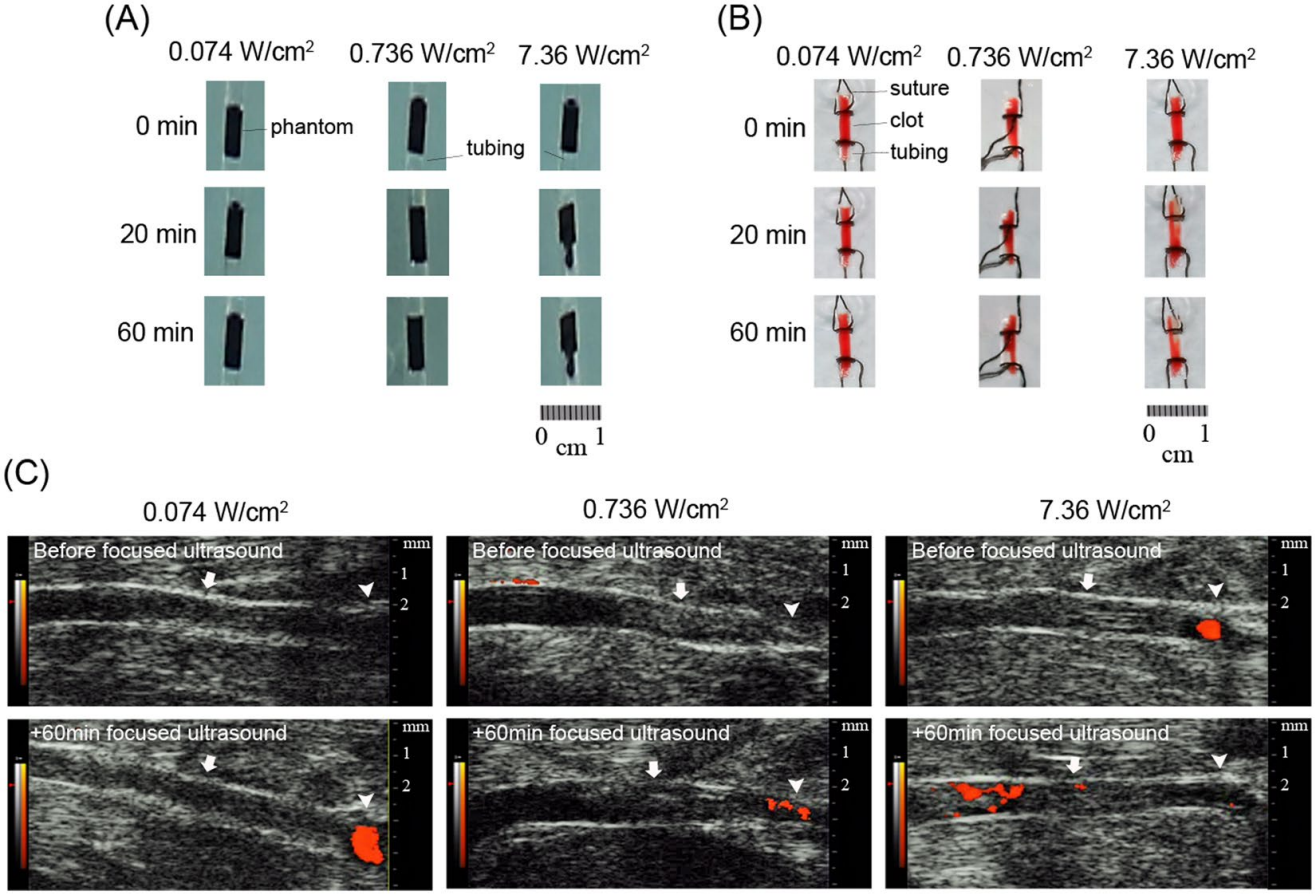
Preliminary study of lysis effect induced by focused ultrasound (FUS) in (**A**) *in vitro* phantom lysis, (**B**) *ex vivo* clot lysis, and (**C**) *in vivo* study under various FUS exposure conditions. *In vitro* (**A**) and *ex vivo* (**B**) lysis tests show noticeable lysis while intermediate or high intensity level is employed (0.736 or 7.36 W/cm^2^). Nevertheless, FUS exposure at any exposure condition cannot effectively induce thrombolytic effect in *in vivo* (**C**) study. In (**C**), arrowhead indicates carotid bifurcation, and arrow indicates thrombotic portion of common carotid artery.

Preliminary studies were done to examine the most suitable intensity level and duration of FUS. The findings of *in vitro* phantom lysis test in different spatial-peak-temporal-average intensity (I_spta_) with different duration (Fig. 1A) showed that under complete occlusion, although no lysis was observed in the lower I_spta_ groups (0.074 and 0.736 W/cm^2^), lysis effect was found in the higher I_spta_ group (7.36 W/cm^2^) after 20-min and 60-min FUS exposure. In the *ex vivo* clot lysis experiment (Fig. 1B), there was noticeable clot lysis only under the intensity of 0.736 and 7.36 W/cm^2^ after 20-min and 60-min FUS exposure. When these conditions were further examined in acute carotid thrombotic occlusion model in rat, there was no recanalization of carotid artery under 0.074 W/cm^2^ for 60 mins, and only partial recanalization was seen under 0.736 and 7.36 W/cm^2^ for 60 mins (Fig. 1C). These preliminary experiments suggested that although FUS could provide physical and mechanical effect for clot lysis, it was less effective when transferring the setups into *in vivo* situations. As 7.36 W/cm^2^ for 60 mins may cause skin burn and the bio-safety threshold of FUS intensity could be a concern by American FDA, the intensity level of 0.736 W/cm^2^ with exposure duration 60 mins was selected for our *in vivo* studies.

### Small-animal ultrasound findings

The B-mode imaging and Doppler change of peak systolic velocity using small-animal ultrasound system were examined in the 11 groups at each time point (Table 1). In all 70 rats, complete occlusion was observed at 2 h after irradiation with implantable wireless light-emitting diode (LED), suggesting the success of the thrombotic occlusion model. In group A, there was no recanalization of the occluded common carotid artery (CCA) and no Doppler signal from 4 h to 7d after 0.45 mg/kg rt-PA bolus treatment. The dose of 0.9 mg/kg rt-PA bolus (group B) yielded recanalization rate from 67% at 4 h to the peak of 83% at 4d but reduced to 50% at 7d, and there was Doppler signal in all vessels at all time points. The use of 0.9 mg/kg rt-PA with 10% bolus + 90% IV fusion (group I) showed better late recanalization rate from 17% at 4 h, 83% at 24 h, 100% at 4d to 83% at 7d than 0.9 mg/kg rt-PA bolus (group B). The treatment of FUS alone (group C) and BNG-1 alone (group D) caused similar results to 0.45 mg/kg rt-PA treatment with a slightly better recanalization rate (17%) in BNG-1 alone at 7d. The 0.45 mg/kg rt-PA treatment when either combined with BNG-1 (group E) or with FUS (group F) showed a similar 50% recanalization rate to 0.9 mg/kg rt-PA alone at 7d. However, 0.45 mg/kg rt-PA 20% bolus + 80% IV fusion + FUS (group H) and 0.9 mg/kg rt-PA bolus + FUS (group J) could give a better late recanalization rate than 0.45 mg/kg rt-PA bolus + FUS (group F) from 4d. Also, 0.9 mg/kg rt-PA bolus + 2 g/kg BNG-1 (group K) could give a better recanalization rate than 0.45 mg/kg rt-PA bolus + 2 g/kg BNG-1 (group E) from as early as 4 h to 7d. Combination therapy of 0.45 mg/kg rt-PA + BNG-1 + FUS (group G) revealed the best optimized recanalization rate from 83% at 4 h reaching 100% at 7d (Table 1).

**Table 1:** Findings of small-animal ultrasound in the 7 treatment groups and 4 comparison groups.

Treatment groups (n = 6)	Number (%) of recanalization* at each time point					PSV (cm/s) in left CCA at each time point					
	After treatment					After irradiation	After treatment				
	4 h	24 h	2 d	4 d	7 d		4 h	24 h	2 d	4 d	7 d
A. 0.45 mg/kg rt-PA bolus	0 (0%)	0 (0%)	0 (0%)	0 (0%)	0 (0%)	0 ± 0	0 ± 0	0 ± 0	0 ± 0	0 ± 0	0 ± 0
B. 0.9 mg/kg rt-PA bolus (n = 8)	5 (63%)	5 (63%)	5 (63%)	6 (75%)	4 (50%)	0 ± 0	26 ± 31	58 ± 36	68 ± 50	58 ± 46	37 ± 55
C. Focus ultrasound (FUS)	0 (0%)	0 (0%)	0 (0%)	0 (0%)	0 (0%)	0 ± 0	0 ± 0	0 ± 0	0 ± 0	0 ± 0	0 ± 0
D. 2 g/kg BNG-1 per os for 7 days	0 (0%)	2 (33%)	2 (33%)	1 (17%)	1 (17%)	0 ± 0	11 ± 21	90 ± 7	97 ± 81	15 ± 30	0 ± 0
E. 0.45 mg/kg rt-PA bolus + 2 g/kg BNG-1	0 (0%)	1 (17%)	2 (33%)	3 (50%)	3 (50%)	0 ± 0	23 ± 40	83 ± 72	75 ± 65	83 ± 79	89 ± 87
F. 0.45 mg/kg rt-PA bolus + FUS (n = 8)	7 (88%)	7 (88%)	7 (88%)	5 (63%)	5 (63%)	0 ± 0	63 ± 28	55 ± 34	76 ± 39	83 ± 63	79 ± 102
G. 0.45 mg/kg rt-PA bolus + FUS + 2 g/kg BNG-1	5 (83%)	6 (100%)	6 (100%)	5 (83%)	6 (100%)	0 ± 0	60 ± 16	86 ± 49	101 ± 45	100 ± 38	97 ± 28
**Comparison groups (n = 6)**											
H. 0.45 mg/kg rt-PA 20% bolus + 80% IV fusion + FUS	3 (50%)	5 (83%)	5 (83%)	5 (83%)	6 (100%)	0 ± 0	62 ± 56	62 ± 12	62 ± 40	57 ± 42	93 ± 31
I. 0.9 mg/kg rt-PA 10% bolus + 90% IV fusion	1 (17%)	5 (83%)	4 (67%)	6 (100%)	5 (83%)	0 ± 0	66 ± 38	84 ± 32	84 ± 37	89 ± 36	76 ± 55
J. 0.9 mg/kg rt-PA bolus + FUS	4 (67%)	4 (67%)	5 (83%)	5 (83%)	4 (67%)	0 ± 0	47 ± 66	54 ± 38	93 ± 66	89 ± 54	55 ± 78
K. 0.9 mg/kg rt-PA bolus + 2 g/kg BNG-1	4 (67%)	4 (67%)	5 (83%)	5 (83%)	5 (83%)	0 ± 0	55 ± 59	49 ± 46	66 ± 38	91 ± 33	88 ± 51

*Recanalization is defined as stenotic degree ≤50% after treatment. CCA = common carotid artery.

**Figure 2:**
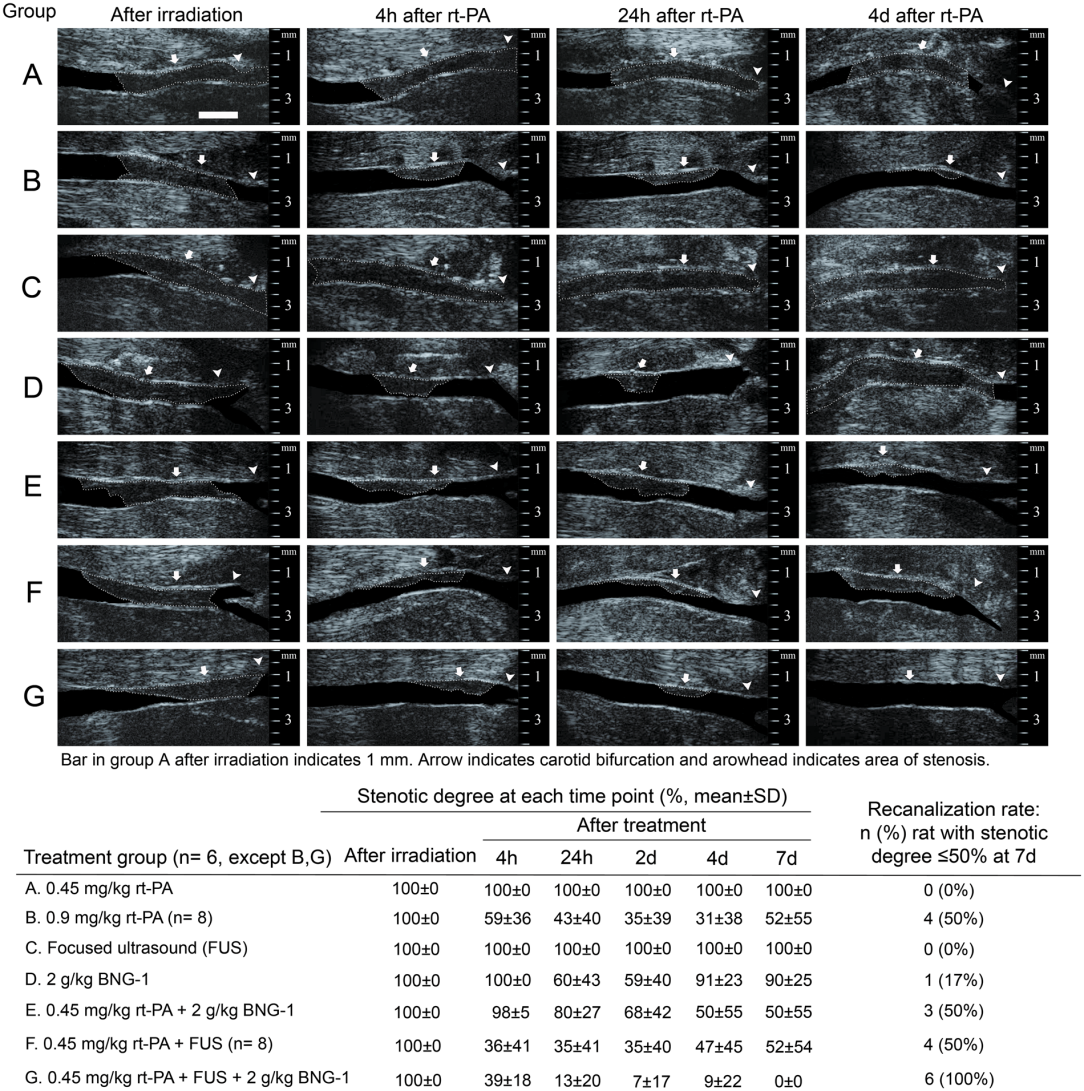
Study of the stenotic degree on small-animal B-mode ultrasound at each time point in each treatment group. Recanalization is found as early as 4 h after rt-PA in groups B, F and G, but improves to 0% stenosis at 7 d only in group G. Groups A and C have persistent occlusion from 4 h to 7 d. Groups D and E have almost complete occlusion at 4 h but recanalize from 24 h with a better recanalization rate in group E at 7 d.

The B-mode imaging using small-animal ultrasound system (Fig. 2) showed 0.9 mg/kg rt-PA (group B), 0.45 mg/kg rt-PA + FUS (group F) and 0.45 mg/kg rt-PA + BNG-1 + FUS (group G) could induce recanalization from 4 h after rt-PA administration. However, the average stenotic degree in groups B and F did not improve much thereafter (4 h to 7d, B: 59 ± 36% to 52 ± 55%, F: 36 ± 41% to 52 ± 54%) but group G improved from 39 ± 18% at 4 h to 0% stenosis at 7d. Groups A (0.45 mg/kg rt-PA) and C (FUS) had persistent occlusion from 4 h to 7d. In the BNG-1 (group D) and BNG-1 + 0.45 mg/kg rt-PA (group E), there was complete occlusion at 4 h, but recanalization was seen from 24 h with a better average stenotic degree in group E at 7d (24 h to 7d, D: 60 ± 43% to 90 ± 25%, E: 80 ± 27% to 50 ± 55%).

### MR image and angiography findings

**Figure 3:**
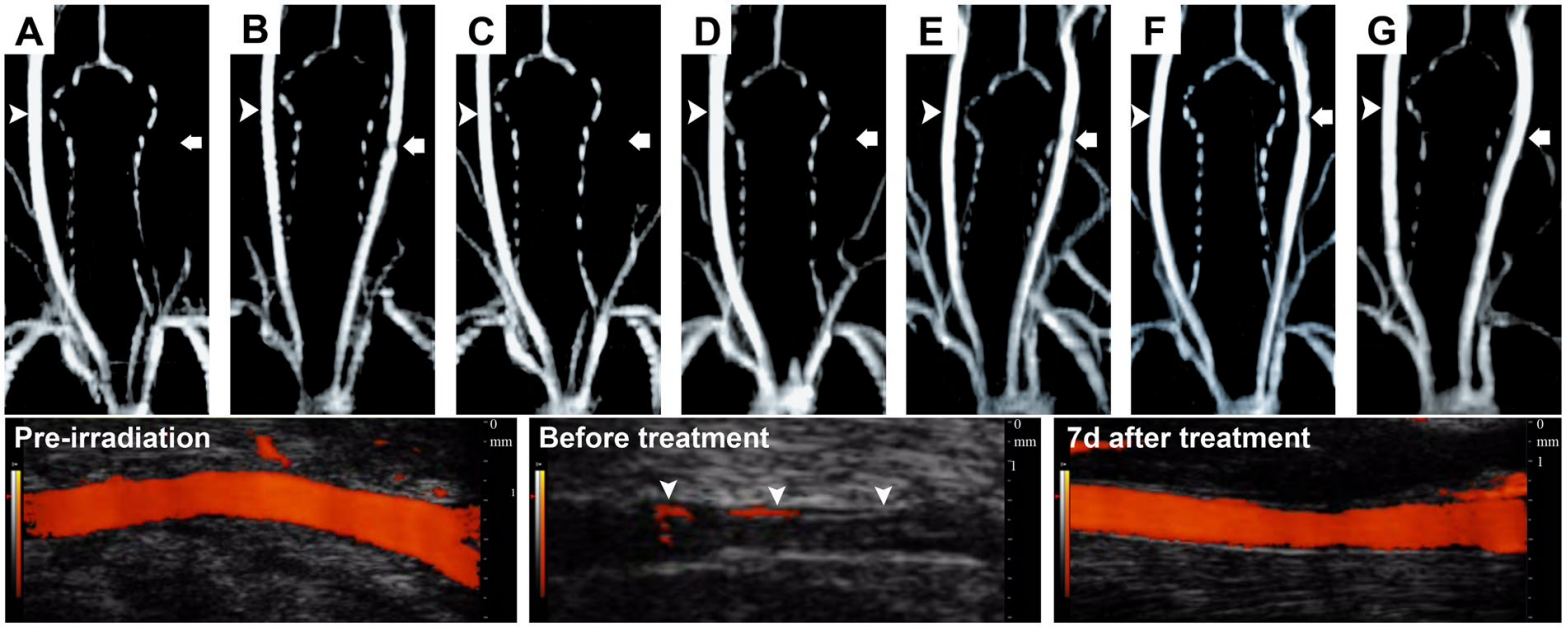
MR angiographic findings at 7d in each treatment group. The left common carotid artery (CCA) is occluded in groups A, C and D. However, left CCA shows some degree of stenosis in groups B, E, and F. In group G, there is complete recanalization with minimal residual stenosis. The carotid ultrasound examination demonstrates the change in group G which shows the normal CCA before LED irradiation (pre-irradiation), after irradiation, complete occlusion of CCA before rt-PA treatment (before treatment, arrowheads indicate vessel wall), and recanalization of occluded CCA at 7d after rt-PA treatment (7d after treatment). In A-G, the arrow indicates the injured left CCA, and arrowhead indicates the normal right CCA.

The MR images did not find any infarction from 1 h to 7 d in all the treatment and comparison groups (data not shown). The MR angiography showed that at 7d after rt-PA, there was occluded left CCA in groups A, C and D (Fig. 3). However, left CCA revealed some degree of stenosis in groups B, E, and F. In group G, complete recanalization with minimal residual stenosis was achieved. In Fig. 3, the carotid ultrasound images demonstrated the change in group G. There was normal CCA before thrombosis induction (pre-irradiation). After irradiation, there was complete occlusion of CCA before rt-PA treatment (before treatment), and the occluded CCA was recanalized successfully at 7d after rt-PA treatment.

### Histopathological findings

**Figure 4:**
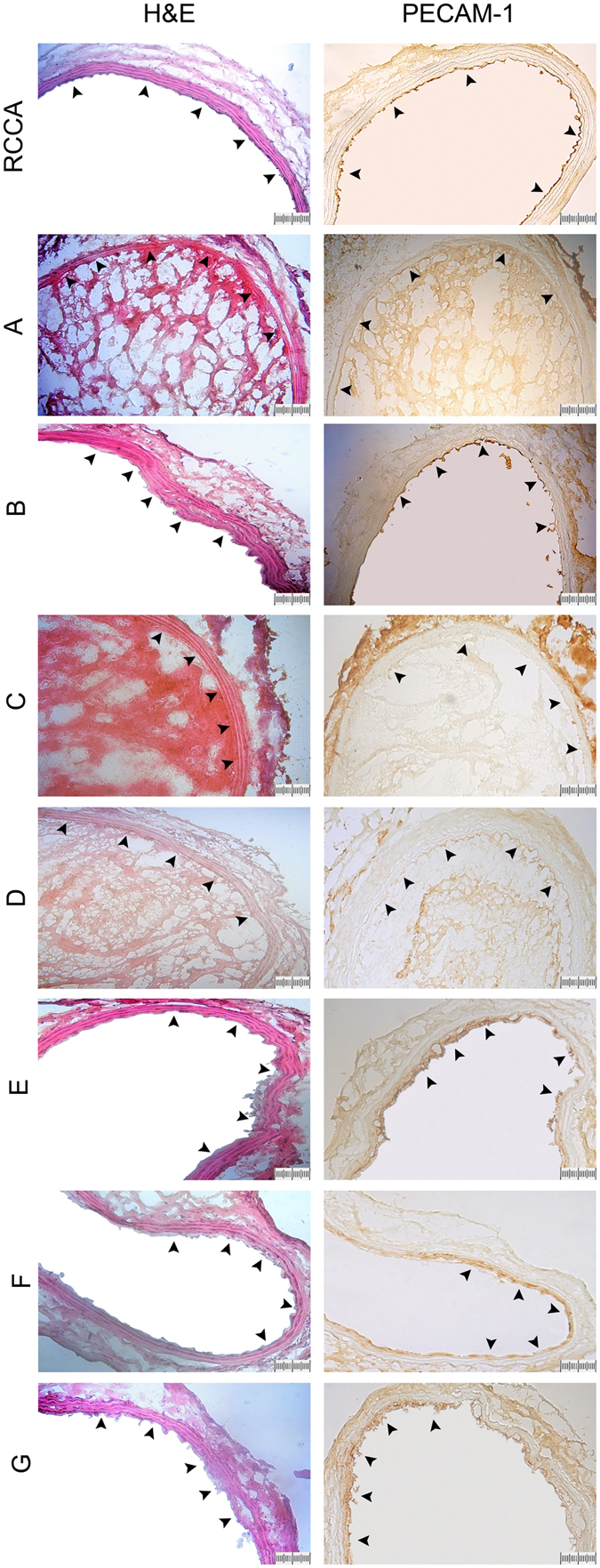
Hematoxylin and eosin (H&E) staining and platelet endothelial cell adhesion molecule-1 (PECAM-1) immunostaining at 7d in each treatment group. The H&E staining shows normal architecture of vessel wall in right CCA (RCCA), and PECAM-1 immunoreactivity is continuously distributed in the endothelium of luminal wall in right CCA. However, in the left CCA of groups A, C and D, the thrombus is present inside the lumen, and the PECAM-1 immunoreactivity is remarkably reduced and distributed discontinuously. In the left CCA of groups B, E, F and G, the thrombus is less present inside the lumen, and the PECAM-1 immunoreactivity is remarkably visible and distributed less discontinuously compared to groups A, C and D. Groups E, F and G show a better endothelial distribution at the luminal wall than groups A, C and D. Arrows indicate the luminal wall of CCA.

The staining of hematoxylin and eosin (H&E) and platelet endothelial cell adhesion molecule-1 (PECAM-1) was examined at 7d after LED irradiation (Fig. 4). The H&E staining showed normal architecture of vessel wall in right CCA, and the PECAM-1 immunoreactivity was present continuously in the endothelium of right CCA. After LED irradiation, thrombosis was induced and the endothelial cells were injured. As seen in the left CCA of groups A, C and D, the thrombus was present inside the lumen, and the PECAM-1 immunoreactivity was remarkably reduced and distributed discontinuously. However, in the left CCA of groups B, E, F and G, the thrombus was less present inside the lumen, and the PECAM-1 immunoreactivity was distributed less discontinuously. Groups E, F and G showed a better recovery of luminal wall and endothelial distribution compared to groups A, C and D.

### Selectivity of BNG-1 on phosphodiesterase (PDE) inhibition

The BNG-1 had inhibitory effects on PDE. The inhibition concentration at 50% (IC_50_) = 24.0 ± 6.2 μg/ml and selectivity = 1.0 was seen on PDE1, IC_50_ = 260.9 ± 10.0 μg/ml and selectivity = 0.09 on PDE2, IC_50_ = 67.9 ± 25.3 μg/ml and selectivity = 0.35 on PDE3, IC_50_ = 301.3 ± 29.9 μg/ml and selectivity = 0.08 on PDE4, IC_50_ = 457.7 ± 40.2 μg/ml) and selectivity = 0.05 on PDE5, IC_50_ = 176.5 ± 24.3 μg/ml and selectivity = 0.14 on PDE6, and IC_50_ = 43.0 ± 1.82 and selectivity = 0.56 on PDE7A.

## Discussion

The present study used acute carotid thrombotic occlusion model induced by LED irradiation which caused vascular endothelial injury and resulted in high re-occlusion rate after rt-PA treatment. The combination therapy of rt-PA with FUS and neuroprotective agent can result in almost complete artery recanalization. Our study highlights a clinically feasible strategy in the synergistic combination of different therapeutic remedies to improve the thrombolytic effect, prevent restenosis after thrombolysis and reduce the rt-PA dose. The use of FUS can help to reduce the dose of rt-PA, and the treatment with BNG-1 may provide benefits with endothelial protection to enhance the anti-thrombotic effect of rt-PA^22^ and prevent restenosis.

Previous *in vivo* studies have reported the combination approach to enhance the effectiveness of rt-PA. The combination of low-dose rt-PA plus recombinant annexin A2 which increases the catalytic efficiency of rt-PA in converting plasminogen to plasmin could enhance thrombolysis efficacy and avoid neurotoxic and hemorrhagic complications^23–25^. Combination treatment with a selective proteasome inhibitor, VELCADE, and low-dose rt-PA could reduce infarction volume with an associated increase in endothelial nitric oxide synthase activity compared to rt-PA alone^26, 27^. Combination treatment of N-acetyl-seryl-aspartyl-lysyl-proline, an endogenously produced circulating peptide with anti-inflammatory and cardioprotective activities, with rt-PA at 4 h after stroke onset substantially reduced infarction volume and neurological deficits without increasing the brain hemorrhage compared with ischemic rats treated with rt-PA alone^28^. Combination treatment of low-dose Niaspan, an extended-release formulation of Niacin (vitamin B3), with rt-PA at 4 hours after embolic stroke could reduce infarction volume, improve neurological outcome and provide neuroprotection^29^. However, these studies used embolic stroke animal model which could cause limited vascular endothelial injury and has low re-occlusion rate after rt-PA treatment. Also, these studies examined the infarction volume but did not directly examine the artery recanalization effect after occlusion.

Sonothrombolysis using transcranial MR-guided FUS in liquefying the clotted blood has been examined in *in vitro* and cadaveric models of intracerebral hemorrhage^30^. An *in vivo* studies using MR-guided FUS to lysis blood clot has been studied in rabbit^19^. Application of MR-guided FUS was also shown to achieve targeted clot liquefaction both in *in vivo* porcine blood clot through a craniectomy model and in *ex vivo* porcine blood clot through human skull model with only marginal increase of temperature in the surrounding tissue^31^. Our previous *in vivo* study also showed the combination use of FUS and MR magnetic targeting could synergistically increase the deposition of therapeutic magnetic nanoparticles in brain across the intact or compromised blood-brain barriers^32^. So it is possible that MR-guided FUS can be applicable in the combination therapy against artery occlusion. A further step in the experiment using larger animals for transcranial approach of FUS may be needed to confirm its feasibility before clinical trial. However, the transcranial loss of focused ultrasound may be human-species or age dependent and should be calibrated prior to exposure. For example, the temporal bone is generally thicker in African or Asian^33^ which dampens FUS penetration through temporal bone compared to Caucasian.

Previous phase II clinical trial demonstrated transcranial sub-MHz ultrasound concomitantly treated with intravenous rt-PA caused a higher rate of cerebral hemorrhages than rt-PA alone^34^. The meta-analysis revealed that sonothrombolysis and sonolysis treatment using transcranial ultrasound could be safe and effective in terms of complete recanalization and favorable functional outcome at three months^35^. However, there are several limitations in these studies such as the lack of adequate sequence generation, no double blindness in randomization and no clinical follow-up, and the small sample size. A recent large phase III clinical trial (CLOTBUST-ER)^14^ showed no significant benefit of sonothrombolysis in acute stroke. Combination therapy of intra-arterial endovascular intervention after medical management such as intravenous rt-PA may improve the outcomes in appropriately selected acute ischemic stroke patients with large artery disease^36^. However, the high medical cost, strict inclusion/exclusion criteria and high bleeding risk have limited its application only in comprehensive stroke center with a well-trained team from different disciplines.

Our study showed when combined with rt-PA and/or FUS, BNG-1 may help to improve the recanalization of acute thrombotic occlusion in carotid artery. The BNG-1 is known to have anti-thrombotic activity which was seen *in vitro* to inhibit arachidonic acid-induced platelet aggregation and *in vivo* to prolong bleeding time in mice^20^. BNG-1 can also inhibit several phosphodiesterase (PDE) isoforms^20^ with potency order of the following rank: PDE1 > PDE7A > PDE3 > PDE6 > PDE2 > PDE4 > PDE5. A potent PDE3 inhibitor, cilostazol^37–39^, is used clinically as an anti-platelet drug to treat ischemic stroke^40, 41^. Intravenous administration of cilostazol nanoparticles was found to ameliorates acute ischemic stroke in cerebral ischemia/reperfusion-induced injury after middle cerebral artery occlusion in mice^42^. As inhibition of PDE7A may be useful in the treatment of inflammatory diseases in immune cells and tissues such as brain or skeletal muscle^43^, the PDE7A inhibition effect of BNG-1 may have therapeutic effect for neurological and inflammation disorders. Our study found there was improved recanalization if BNG-1 was given in the rats treated with 0.45 mg/kg rt-PA bolus. This recanalization could be seen at as early as 4 h if BNG-1 was added in the rats treated with 0.45 mg/kg rt-PA bolus + FUS or in the rats treated with 0.9 mg/kg rt-PA bolus. In future studies, the dose of BNG-1 and the combination therapy with different remedies should be well designed to improve the recanalization effect.

The present study was performed under certain limitations. First, although FUS is suggested to be safer than transcranial ultrasound which used planar unfocused exposure^18^, the bleeding risk of FUS still needs to be carefully investigated before clinical application when rt-PA is concurrently administered. Second, although *in vivo* studies^19, 31^ have shown the potential of FUS for blood clot lysis, the optimized parameters and the nature of ultrasound, the risk of bleeding, the accurate transcranial guiding of FUS on thrombotic artery, the potential effect on the adjacent parenchyma, and the chemical and toxicity profiles of resulting lysate particles need further assessment before clinical practice. Third, the present study showed bolus injection of 0.9 mg/kg rt-PA induced better early recanalization rate at 4 h but had high restenosis rate (group B) than 0.9 mg/kg rt-PA 10% bolus + 90% IV fusion (group I). The high restenosis rate of bolus injection can be improved after the combination treatment with FUS (group J) or BNG-1 (group K). However, bolus injection is distinct from clinical practice using intravenous infusion within one hour and its feasibility to carry out in acute ischemic stroke treatment needs further investigation.

In conclusion, our study showed the potential of combination therapy using thrombolytic agent, FUS and neuroprotective agent in the recanalization treatment of acute carotid artery thrombotic occlusion. It is possible that combination therapy with different remedies may offer a novel treatment strategy to extend the therapeutic time window to become an alternative therapy other than mechanical thrombectomy.

## Methods

### Study design

**Figure 5:**
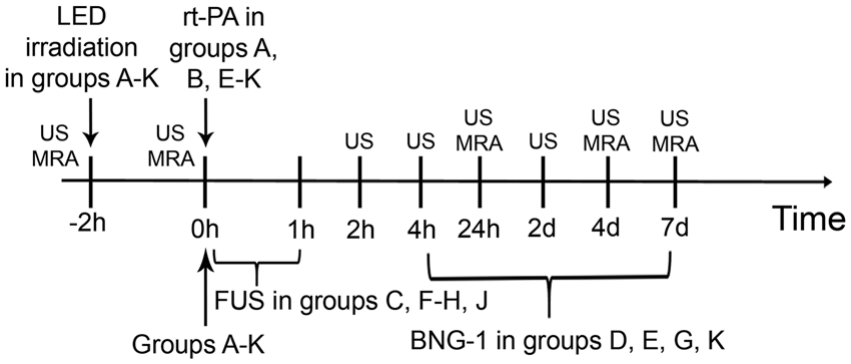
Schema of the experimental design. Seven treatment groups (n = 6–8 in each group) are examined including (A) intravenous bolus injection of 0.45 mg/kg rt-PA (low-dose), (B) intravenous bolus injection of 0.9 mg/kg (standard-dose, n = 8), (C) sonothrombolysis with focused ultrasound (FUS) alone, (D) oral administration of 2 g/kg BNG-1 alone for 7 days, (E) combination of A and D, (F) combination of A and C (n = 8), and (G) combination of A, C and D. Another 4 groups are made for comparison including (H) 0.45 mg/kg rt-PA 20% bolus + 80% IV fusion + FUS, (I) 0.9 mg/kg rt-PA with 10% bolus + 90% intravenous fusion, (J) combination of B and C, (K) combination of B and D. US = small-animal carotid ultrasound study. MRA = magnetic resonance angiography study.

Seven treatment groups (n = 6–8 in each group) were examined including (A) intravenous bolus injection of 0.45 mg/kg rt-PA (low-dose), (B) intravenous bolus injection of 0.9 mg/kg (standard-dose, n = 8), (C) sonothrombolysis with FUS alone, (D) oral administration of 2 g/kg BNG-1 alone for 7 days, (E) combination of A and D, (F) combination of A and C (n = 8), and (G) combination of A, C and D. Another 4 groups were made for comparison including (H) 0.45 mg/kg rt-PA 20% bolus + 80% IV fusion + FUS, (I) 0.9 mg/kg rt-PA with 10% bolus + 90% intravenous fusion, (J) combination of B and C, (K) combination of B and D. rt-PA was given 2 h after LED irradiation and then FUS treatment was given for 1 h to insure the combination treatment be completed within 3 h after thrombotic occlusion (Fig. 5), similar to the treatment time window of rt-PA. The rat was randomly assigned to one of the 11 treatment group. The investigator who did the LED irradiation was blind to the therapeutic strategy.

### Small-animal carotid ultrasound

Carotid ultrasound (Vevo 2100, Visual Sonics, Toronto, Ontario, Canada) was equipped with a 40-MHz transducer (MS550D) with high resolution B-mode, color Doppler energy mode (Power mode) and pulse-wave Doppler. The ultrasound was performed before LED irradiation, before rt-PA treatment (groups A, B, E-K) or at 2 h after LED irradiation (groups C, D) and at 2 h, 4 h, 24 h, 2 d, 4 d, and 7 d after rt-PA treatment. For groups C and D with no rt-PA treatment, 2 h after LED irradiation was set as 0 h (Fig. 5).

### Animal

Male Sprague-Dawley rats weighted 400–450 grams and aged 14–16 weeks old were obtained from National Animal Center. Efforts were made to minimize suffering, reduce the number of animals used, and utilize alternatives to *in vivo* techniques, if available. All procedures were performed in accordance with the National Institute of Health Guide for the Care and Use of Laboratory Animals (NIH Publications No. 80-23) revised in 1996. The animal subject review board of Linkou Chang Gung Memorial Hospital provided formal approval to conduct the experiments described.

### Acute carotid thrombotic occlusion model

The acute thrombotic occlusion model was created in common carotid artery according to our previous study^44^. Rats were first anesthetized with 3% isoflurane in a mixture of air with nitrous oxide and oxygen (70%/30%) for induction and then maintained at 1.5–2% isoflurane during the whole experiment. Left femoral vein was exposed, and a polyethylene tube (PE-10, Becton, Dickinson and Company, Franklin Lakes, New Jersey, USA) was inserted into the vein for rose bengal injection. The left CCA was dissected out, laid in the ditch on the LED lens and fixed by suture. After the implantation of left CCA, the surgical wound was sutured, and rats were placed in prone position on the transmission coil of external controller. By powering on external controller, 1 mL of 60 mg/kg rose bengal (Sigma-Aldrich, St. Louis, MO, USA) was administrated intravenously, and the left CCA was irradiated with the implanted LED (540 nm, 4.6–4.8 mW/cm^2^) for 2 h first, shut off for 30 mins, and then irradiated again for another 2 h. After irradiation, LED was removed and wound was sutured.

### Focused ultrasound

**Figure 6:**
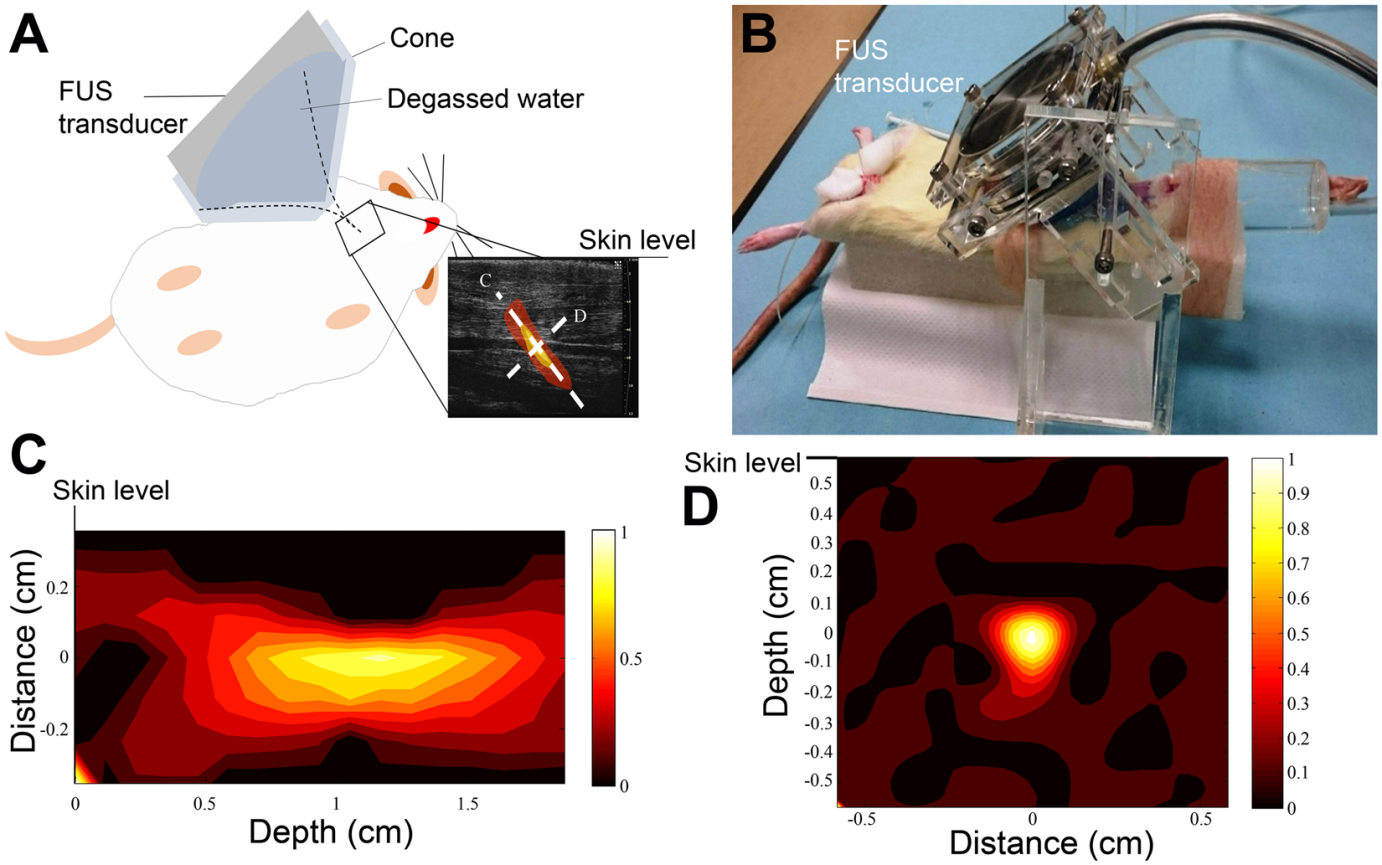
Design of focused ultrasound (FUS) exposure to left common carotid artery of rat. (**A**) Schema of the application of FUS in rat. (**B**) The actual picture of FUS application in rat. (**C** and **D**) The depth of FUS exposure below the skin level.

To obtain the optimal power of FUS, various exposure parameters of FUS were examined (Fig. 1). A concentric and confocally arranged dual-frequency FUS transducer was employed (Fig. 6) with the center frequency of 0.55 MHz and 1.1 MHz, respectively. The center frequency of the inner and outer ceramics was 1.1 MHz (Sonic Concepts, USA; ceramic diameter = 64 mm, radius of curvature = 63.2 mm, frequency = 1.1 MHz). The FUS transducer was driven by a power amplifier (500–009, Advanced surgical system, Tucson, AZ, USA) connected with a function generator (33220A, Agilent, Palo Alto, CA, USA). The therapeutic transducer was attached to a stage and oriented downwards from above the left CCA at an angle of 60 degrees, and was coupled to the skin with ultrasound gel. The focal spot generated by the transducer was carefully measured by a polyvinylidene-difluoride-type (PVDF) hydrophone (HNP-0400, ONDA, Sunnyvale, USA). During the pressure distribution measurement, the therapeutic transducer was attached to a 3-axis position stage with the moving step of 0.5 mm. The measured pressure field of ultrasound transducer in the −3 dB dimensions was about 1.5 mm wide and 8 mm long at half-amplifier (width at −6 dB points). FUS pulse sequence was examined to find the largest power able to cause thrombolysis without injury to perivascular soft tissue. The input electric power of 1.2–12.1 W was attempted. According to our preliminary *in vivo* study, burst-mode ultrasound with a burst length of 10–100 ms, pulse-repetition frequency of 1 Hz, and exposure duration of 20–60 mins was attempted. The equivalent intensity (I_spta_) was ranged in 0.074–7.36 W/cm^2^, with the peak FUS exposure pressures ranged from 0.47–1.5 MPa.

### *In vitro* and *ex vivo* assessment of lysis effect induced by focused ultrasound

*In vitro* phantom lysis and *ex vivo* clot lysis experiments were conducted to assess the lysis efficiency of FUS to obtain the optimal thrombolytic effect in exposure parameter selection. In *in vitro* phantom lysis experiment, glue and graphite were mixed in the PE-50 tube (i.d. 0.58 mm). In *ex vivo* clot lysis experiment, blood clots were formed by natural coagulation of blood sample. Briefly, whole blood was drawn slowly from femoral artery of rats into a PE-50 tubing (PE-50, Becton, Dickinson and Company, Franklin Lakes, New Jersey, USA) and incubated at 37 °C for 2 h and then stored at 4 °C for another 22 h. The clots were sunk in the tank filled with degassed water, and placed directly on the focused point of transducer. The selected spatial-peak-temporal-average intensity (I_spta_) was 0.074, 0.736 and 7.36 W/cm^2^ which combined input electric power of 1.2 and 12 W (corresponding to 0.469 and 1.486 MPa of acoustic pressure) with 10 and 100 ms burst length. The other static parameters were 1 Hz pulse-repetition frequency and 60-min exposure duration.

Carotid thrombotic occlusion model was employed to evaluate the lysis efficiency of FUS. Rats were anaesthetized with 3% isoflurane and maintained at 1.5%. After thrombotic occlusion by LED irradiation, rats were then laid in a supine position (hair removed from the bottom of thigh) on a platform located atop a water tank, and exposures were conducted through a water-coupling cone mount on the FUS transducer (Fig. 6). After clot position confirmation via small-animal ultrasound, alignment of the FUS beam with the thrombus in CCA was achieved using pulse echo measurements with therapeutic transducer. For single location study, small-animal ultrasound system was employed to quantify clot erosion. Volumetric images along 30 mm longitudinal segments were acquired pre- and post-treatment with the eroded clot boundary being delineated based on the lower echogenicity of the eroded region relative to the intact clot.

### rt-PA and BNG-1 preparation and administration

rt-PA was obtained as lyophilized powder (Actilase, Boehringer Ingelheim Pharma GmbH & CO. KG, Ingelheim, Germany) and mixed with sterile water per manufacture’s instruction. BNG-1 was provided as dried powder (Braingenesis Biotechnology CO., LTD, Taipei, Taiwan) and dissolved in saline as vehicle. The oral administration dose of BNG-1 was 2 g/kg at a volume of 10 ml/kg^20^.

### Determination of thrombolytic efficiency

The thrombolytic efficiency was examined by small-animal ultrasound. The thrombus length and diameter stenotic degree of CCA were examined from B-mode images. The stenotic degree was determined: stenotic degree (%) = (1 − D_T_/D_L_) × 100%. Where D_L_ was the original lumen diameter and D_T_ was the stenotic lumen diameter at the portion with the largest thrombus formation. Recanalization was defined if the stenotic degree was reduced to ≤50%. The blood flow change of peak systolic velocity (PSV, cm/s) was measured by pulse-wave Doppler. The contralateral right CCA was used as control.

### Magnetic resonance (MR) image and angiography

MR image and angiography using 7T MR imaging system (Clin Scan 70/30 USR, Bruker, Rheinstetten, Germany) was studied to examine the presence of cerebral infarction and hemorrhage and the degree of intracranial/extracranial artery stenosis/occlusion. The rats were anaesthetized and maintained with 1.5% isoflurane. According to our previous method^22^, T1-weighted, T2-weighted, apparent diffusion coefficient (ADC), maximum intensity projection (MIP) of susceptibility weighted imaging (SWI) and time-of-flight (TOF) of MR angiography were studied. Transverse slices were acquired with a fast, low-angle shot using the following parameters: TR = 22 ms, TE = 4.87 ms, pulse angle = 90 degrees, field of view = 55 × 42 mm^2^, and matrix size = 58 × 256. The MR angiogram was examined before LED irradiation, before rt-PA treatment or at 2 h after LED and at 24 h, 4 d and 7 d after rt-PA treatment. The contralateral right CCA was used as control.

### Histologic and immunohistochemical study

At 7 d after completion of the final MR imaging, rats were anesthetized and euthanized. The left and right CCAs were removed and washed with phosphate buffer solution, dehydrated on dry ice, and store at −80 °C for 24 h. Each CCA was embedded in Optimal Cutting Temperature (Tissue Tek 4583, Sakura Finetek USA, Inc., Torrance, California, USA) and stored at −80 °C for another 12 h. Transverse section of 10 μm was cut at a microtome-cryostat. Sections were stained with hematoxylin and eosin (H&E) to evaluate the thrombus formation and the architecture of vessel wall. For immunohistochemical staining, the sections were fixated in ice-cold acetone, incubated overnight at 4 °C with the primary antibody (anti-PECAM-1, AbD serotec, Dusseldrof, Germany) and then reacted for 1 h with a universal immunoperoxidase polymer (Histofine, Cosmo Bio, Carlsbad, California, USA) at room temperature. Development of color was achieved by exposure for 3 mins to the DAB chromogen system (DAKO, Carpinteria, CA, USA).

### Phosphodiesterase Inhibition Assays

PDE1 partially purified from bovine heart was used. BNG-1 or vehicle was incubated with 13 μg enzyme, 1.0 μM cold AMP with [3H]-cAMP and CaCl_2_/calmodulin in Tris buffer pH 7.5 for 20 minutes at 30 °C. The reaction was terminated after 2 minutes of boiling. The resulting AMP was converted to adenosine by addition of 10 mg/ml snake venom nucleotidase and further incubation at 30 °C for 10 minutes. Unhydrolyzed cAMP was bound to AGI-X2 resin, and the remaining [3H]-adenosine in the aqueous phase was quantitated by scintillation counting. The assays of PDE2, PDE3, PDE4, PDE5 and PDE6 were similar to PDE1 with different enzyme source, enzyme amount, cold cAMP/cGMP, [3H]-cAMP/[3H]-cGMP and quantitation. For PDE7A assay, human recombinant PDE7A expressed in insect Sf9 cells were used. BNG-1 and/or vehicle was preincubated with 108 ng/ml enzyme in Tris-HCl buffer pH 7.2 for 15 minutes at 25 °C. The reaction was initiated by addition of 100 nM fluorescein labeled cAMP for another 30-min incubation period and terminated by addition of IMAP binding solution. IMAP complexed with phosphate groups on nucleotide monophosphate generated from cyclic nucleotides through PDE activity. Determination of the amount of complex formed was read spectrofluorimetrically at 470 nm/525 nm.
